# The MTIST platform: a microbiome time series inference standardized test

**DOI:** 10.21203/rs.3.rs-4343683/v1

**Published:** 2024-05-08

**Authors:** Jonas Schluter, Grant Hussey, João Valeriano, Chenzhen Zhang, Alexis Sullivan, David Fenyö

**Affiliations:** NYU Langone Health; NYU Grossman School of Medicine; Centre Interdisciplinaire de Nanoscience de Marseille, Aix-Marseille Université; NYU Grossman School of Medicine; NYU Grossman School of Medicine; NYU Langone Health

**Keywords:** microbiome, ecology, ecological interactions, machine learning, ecosystem inference

## Abstract

The human gut microbiome is a promising therapeutic target, but interventions are hampered by our limited understanding of microbial ecosystems. Here, we present a platform to develop, evaluate, and score approaches to learn ecological interactions from microbiome time series data. The microbiome time series inference standardized test (MTIST) comprises: a simulation framework for the *in silico* generation of microbiome study data akin to what is obtained with quantitative next-generation sequencing approaches, a compilation of a large curated data set generated by the simulation framework representing 648 simulated microbiome studies containing 18,360 time series, with a total of 2,182,800 species abundance measurements, and a scoring method to rank ecological inference algorithms. We use the MTIST platform to rank five implementations of microbiome inference approaches, revealing that while all algorithms performed well on ecosystems with few species (3 and 10), all algorithms failed to infer most interaction in a large ecosystem with 100 member species. However, we do find that the strongest interactions within a large ecosystem are inferred with higher success by all algorithms. Finally, we use the MTIST platform to compare different microbiome study designs, characterizing tradeoffs between samples per subject and number of subjects. Interestingly, we find that when only few samples can be collected per subject, ecological inference is most successful when these samples are collected with highest feasible temporal frequency. Taken together, we provide a computational tool to aid the development of better microbiome ecosystem inference approaches, which will be crucial towards the development of reliable and predictable therapeutic approaches that target the microbiome ecosystem.

## Introduction

The human gut microbiome comprises a diverse ecosystem of bacteria, fungi, archaea, and viruses, promising vast potential for novel therapeutic applications. While the multi-kingdom diversity of the gut is only beginning to be explored^1–8^, a majority of microbiome research has focused on bacterial communities because their large population numbers and genotypic diversity facilitate the application of next-generation sequencing approaches for microbiome profiling. In patients suffering from a wide range of diseases, bacterial population compositions have been found to deviate from healthy controls, sometimes with a striking pattern specific to the disease state.^9–11^ While cause-and-effect relationships between disease and bacterial microbiota are rarely clear, first interventions are being approved that seek to manipulate the microbial ecosystem,^12,13^ but our understanding of ecological interactions in the microbiome remains limited.

Bacterial ecosystems have been characterized by computing correlation networks between microbial abundances.^14–21^ However, co-occurrences and correlations tell us little about how two bacterial taxa interact.^22–24^ For example, two taxa that consume the same nutrients may show a positive correlation even though they are competing for the nutrients. Likewise, if one bacterium benefits from the metabolites produced by another, these two species may also show positive correlations even though their ecological interaction is categorically different from the first example^1^. Understanding ecological interactions will be crucial for microbiome-targeted interventions. E.g. microbial interactions within the microbiome could explain why fecal microbiota transplants (FMTs) fail to reliably manipulate ecosystems in the microbiome^25^, even in autologous FMTs where the transplant material is sourced from the recipient themselves prior to a microbiome-injuring therapy^26^.

Taxon richness and complexity of the bacterial gut microbiota make understanding this ecosystem challenging^24^ and hamper our ability to design rational and targeted interventions. Mathematical models from community ecology have been developed to bridge the theoretical gap when studying the gut microbiome.^27–29^ In these models, differential equations describe microbial population dynamics,^27,30,31^ and sometimes include terms to model diet-derived nutrient concentrations over time.^30,32^ The family of generalized Lotka-Volterra (gLV) models considers that each taxon (e.g. species in the model) may interact with itself and with other species,^27,33^ i.e., they capture the elementary pairwise interactions that drive ecological dynamics: mutualism (+/+), competition (−/−), exploitation (+/−), commensalism (+/0), and amensalism (−/0).^27,33,34,34,35^ Focusing on such pairwise interactions means that more complex ecological scenarios will be missed (e.g., when two species interact only when a third one is present, or when non-instantaneous, delayed interactions are considered^36^), as well as some indirect effects that arise when interactions between species and their nutrients are modeled explicitly.^30,31^ Despite these well-known limitations of pairwise interaction models, they have been the dominant choice for the development of algorithms that seek to infer ecological interactions from next-generation sequencing data^1,37–47^ thanks to their causal interpretability.^24^ Microbial abundance profiles recorded over time from the same animal or human donor or during *in vitro* experiments provide the data that can be used to estimate pairwise interaction strengths in gLV systems using regression techniques if total abundances (i.e., concentrations of bacteria), are measured.^1,24,31,43,44,47–49^

Many related inference algorithms have been recently proposed to estimate gLV coefficients and thereby quantify ecological interactions.^1,37–46^ Despite this proliferation of inference tools, no unified method exists to assess inference algorithm performances. Some studies use numerical simulations of the gLV differential equations with the inferred coefficients to compare the simulated time series to observed data by calculating the root mean squared errors or the Bray-Curtis dissimilarity between simulated and observed communities.^37,38^ However, while such measurements provide a goodness of fit metric, they cannot conclude whether an algorithm correctly inferred the unknown real interactions. Quantifying how taxa are most likely to interact with each other is the main advantage of model-based inference approaches.^1,24^ An alternative scoring approach can measure the performance of an inference algorithm on this task by simulating microbial time series from gLV systems with known coefficients, captured by their corresponding community matrices for a comparison with the pairwise interactions that the algorithm inferred from data. A major limitation of this approach is that there is currently no unified test dataset as a standard to benchmark inference algorithms. The field of machine learning has long been using standardized test datasets to compare learning tools; most famously, the MNIST dataset that contains annotated examples of handwritten digits is used to rank classification algorithms.^50^

Taking inspiration from the utility of the MNIST dataset to score a broad range of learning algorithms, we present MTIST, a platform for the standardized evaluation, scoring, and comparison of microbiome ecosystem inference tools. We provide a scoring system which focuses on the nature of interaction types between two species, i.e. the sign of their interaction coefficients, and we evaluate five implementations of gLV-inference algorithms, contrasting their ability to capture the correct nature of interspecies interactions and analyze optimal microbiome study designs to unravel ecological interactions in the microbiome.

## Results

**Figure 1:**
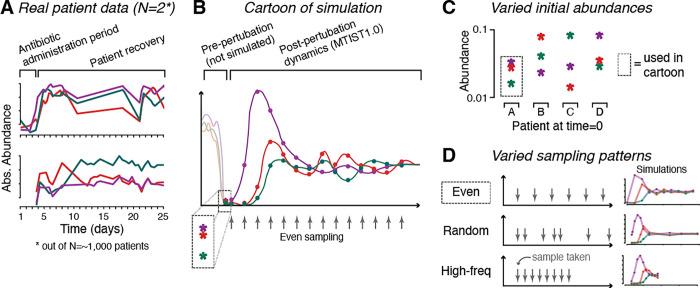
The MTIST simulation framework. A. Total abundances of 3 taxa measured in stool samples from two patients over time following strong microbiome perturbation (N=2 out of approx. 1,000, Liao et al. 2021). B-D. The simulation framework developed to generate MTIST1.0 B. Cartoon of one simulation of three species over time representative of the MTIST data. C. Initial species abundances vary in each simulated time series, akin to different study subjects with different perturbations. D. MTIST implements three study designs via different sampling schemes: evenly distributed (“even”), randomly with varying time differences between samples (“random”), or sequentially with the maximum frequency (“high-freq”).

**Figure 2:**
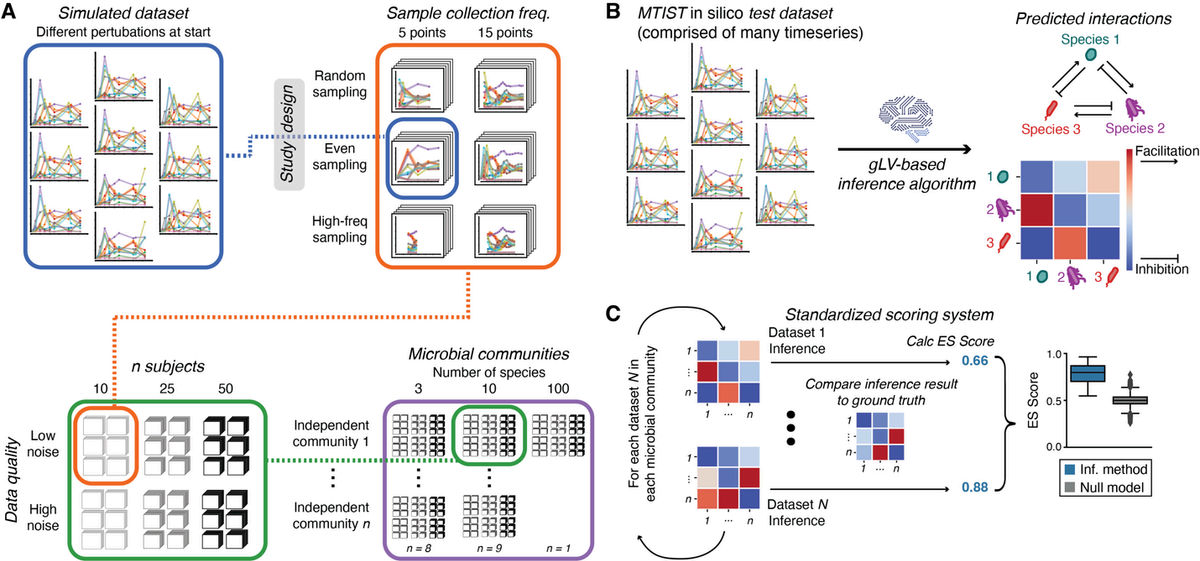
The MTIST platform. **A.** Individual time series sharing the same underlying community matrices are simulated with different initial perturbations, i.e. initial species abundances, three different sampling schemes (random, even, high-freq), two different sample numbers per time series, and three different numbers of independent time series (“subjects”). This is repeated for each of the 18 *in silico* bacterial communities, yielding 648 simulated microbiome studies. **B.** Ecosystem inference algorithms ingest the time series data from each of the 648 MTIST test datasets one at a time to infer the underlying bacterial community matrix ground truth; a representative workflow with a 3-species example is shown here. **C.** Schema of the ES score calculation to rank how algorithms performed on each inference task. Inference results are calculated for all datasets corresponding to one specific example of the 18 bacterial community matrices (ground truths); the focal inference approach applied to data set #1 and #*N* here achieved ES scores of 0.66 and 0.88, respectively. Scores are then aggregated and compared to a null model or other algorithms, on subsets or the entire MTIST1.0 data.

Our simulation framework implements generalized Lotka-Volterra (gLV) equations that were extended to include additional factors that influence the simulated time courses (**Supplementary Methods**). They include biological events such as migration, perturbations associated, for example, with host intake of foods, variation in initial community compositions which is expected in a diverse patient population, especially following the ingestion of antibiotics (Fig. 1A–C), and different study designs (Fig. 1D). This simulation framework for generating in silico microbiome study data is provided open source via GitHub alongside a large, curated data set of simulated microbiome time series and tools for evaluating and ranking microbiome ecosystem inference algorithms (github.com/jsevo/mtist). The implementation of our framework was inspired by gut microbiome dynamics observed during the assembly of the infant gut microbiome^1,27^ and recovery dynamics during secondary succession after antibiotic perturbation observed in hospitalized cancer patients (Fig. 1A).^51–56^ The collection of test datasets contained in MTIST1.0 was generated by taking samples from the simulated ecosystems in a way that mimics three different microbiome study designs (Fig. 1D). Together, MTIST1.0 includes 648 simulated microbiome studies, comprised of 18,360 microbiome time courses and 2,182,800 simulated species abundance measurements (Fig. 2).

We designed a simple score that quantifies ecological insights obtained by an inference tool evaluated on the MTIST platform (Fig. 2B, C). One important purpose of ecological inference is to yield engineering insights, e.g., to predict if a fecal microbiota transplant is likely to take hold within a recipient or how the abundance of beneficial or harmful microbes could be supported or reduced, respectively. Attempts to do so via manipulating ecological network effects require the correct detection of signs of ecological interactions between species. Therefore, the ES score compares known interaction coefficients between all species pairs with the inferred coefficients normalized by the number of interactions. When evaluating a focal inference tool, an ES score is calculated for each dataset contained within MTIST (Fig. 2A, blue box), each representing a hypothetical microbiome study (Fig. 2B, left), by using the inferred community matrix and the community matrix used for simulation (Fig. 2B, right). This yields a distribution of ES scores for the focal inference tool, which can be compared to the results of other algorithms as well as a baseline expectation, obtained by generating inference results at random (Fig. 2C).

**Figure 3:**
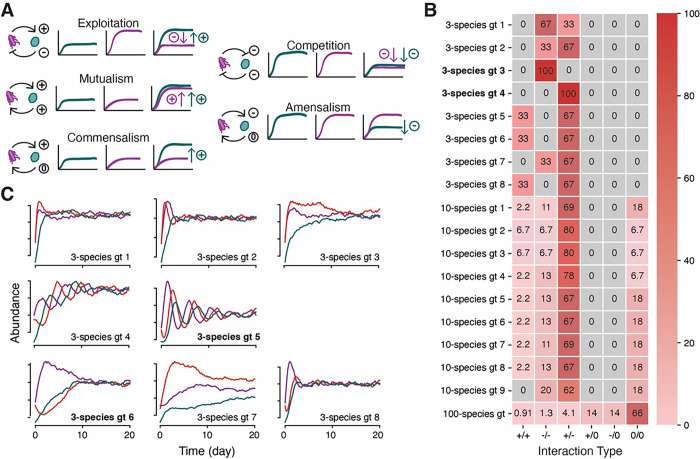
Different synthetic communities capture major types of pairwise interactions. A. Cartoon of the ecological effects of each interaction type in the simulated ecosystems. For example, in exploitation, the exploiting blue species reaches a higher capacity in presence of the orange species compared with growth on its own, while the exploited orange species reaches lower capacity when together. B. Heatmap of the percentages of interaction type categories across the 18 different community matrices used as the ground truths for inference scoring. C. Despite identical interaction type category frequencies, different community matrices yield different dynamics. Here, example time series from each 3-species community are shown. Despite “3-species gt 5” and “3-species gt 6” sharing the same composition of interaction types, they yield different community dynamics.

In the MTIST evaluation workflow, each algorithm is ranked by how well it uncovers the correct community matrix, i.e. the species-by-species interaction coefficients, used to generate the MTIST1.0 datasets. We designed the bacterial communities to contain diverse ecological interactions in different proportions and ecosystems, representing, for example, different habitats or host environments. In total, we used 18 community matrices as the to-be-inferred ecological ground truths: eight 3-species communities, nine 10-species communities, and one 100-species community. They contain different fractions of non-interacting pairs (0/0) and different frequencies of elementary pairwise interactions (Fig. 3). In the gLV systems, each pairwise interaction describes how the respective species modify each other’s growth rates by altering ecological capacities (Fig. 3A). The pairwise interactions, combined with non-differentiable noise (**Figure S1**), determine the evolution of species abundances over time in the MTIST simulation framework. We summarize the interaction type distributions for each of the 18 communities (Fig. 3B) and show representative simulation results (Fig. 3C). Different community matrices yield characteristic dynamics. For example, in a small community comprising species that interact purely exploitatively, characteristic oscillations arise before the community converges towards its equilibrium (Fig. 3C, label “3-species gt 4”); on the other hand, when most interactions are competitive, convergence to equilibrium follows smooth trajectories (Fig. 3D, label “3-species gt 3”). In the eight 3-species communities, all species interact and each of the fundamental interaction types (exploitation, competition, and mutualism) are represented in at least one of the communities; in the nine 10-species communities, 82–93% of species interactions are nonzero, and in the 100-species community 44% of all species interactions are nonzero. All five interaction types are represented in the 10- and 100-species communities.

**Figure 4:**
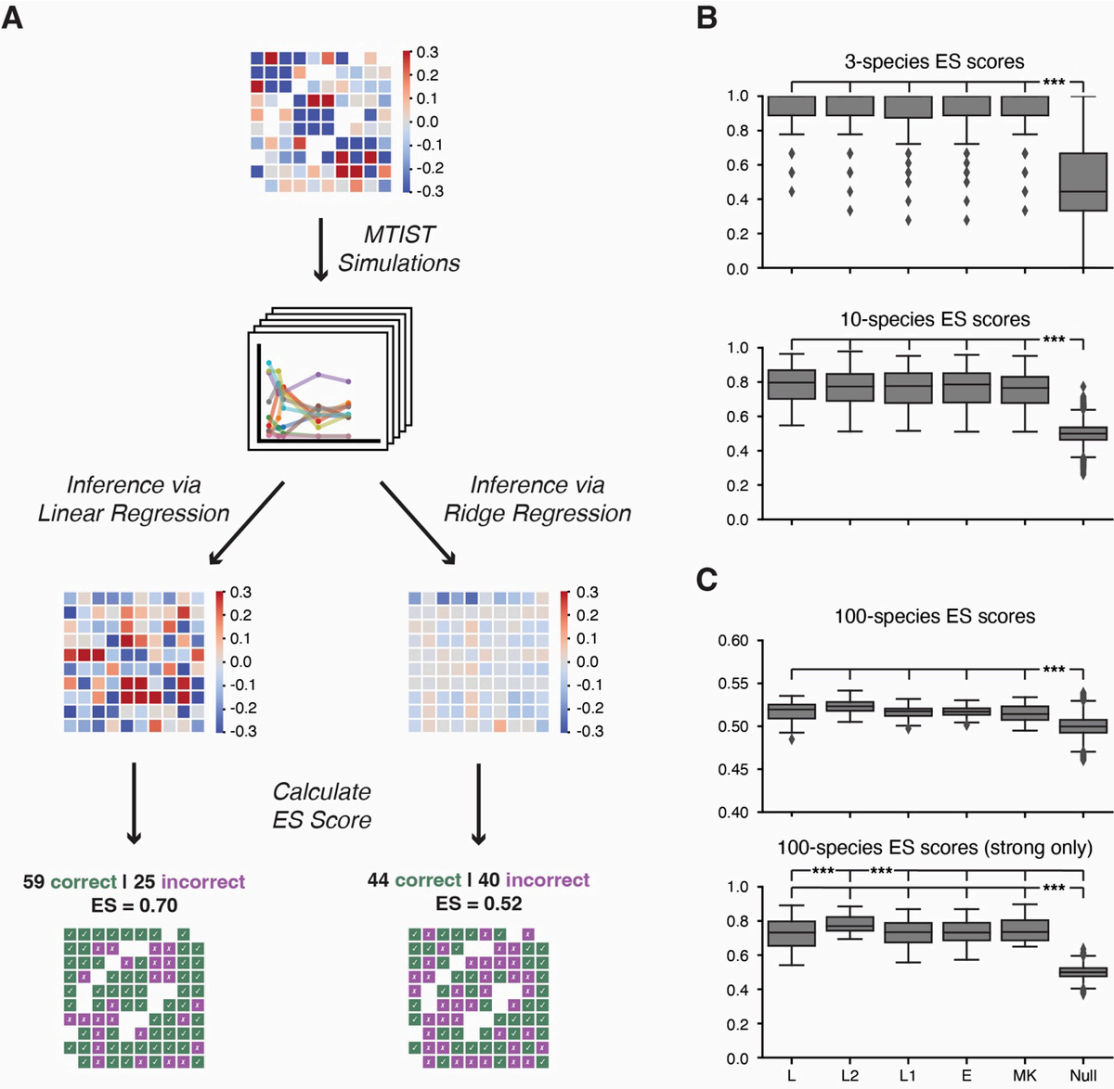
ES score allows for the performance of five different algorithms to be compared. A. In this example, inference is completed using a linear regression and ridge regression-based algorithm on MTIST dataset id 200 (top). For each raw inference result from each regression (middle), the number of correct and incorrect sign inferences were tallied and ES score was calculated (bottom). By counting the points and scaling the sum between 0 and 1 (see Supplementary Methods), ES score is calculated. Since the ES score of linear regression is greater, due to 30 more net correct sign inferences, linear regression can be said to perform more accurate inference for this specific dataset with its unique set of conditions. B. Stars indicate the result of a Wilcoxon signed-rank test between each inference method (L, L2, L1, E, or MK) and the corresponding null distribution (far right). All inference methods generally succeed in inferring 3- and 10-species communities. C. Top row of stars indicate the result of a Wilcoxon signed-rank test between L/L2 and L2/L1. Second row of stars indicate the result of a Wilcoxon signed-rank test between each inference method (L, L2, L1, E, or MK) and the corresponding null distribution (far right). While 100-species communities are a challenge for inference algorithms, all algorithms perform generally well at inferring “strong” interactions (where |βij| > 0.25).

Using the MTIST1.0 simulation data, we next compared five different ecosystem inference approaches. We implemented four inference algorithms that learn interaction coefficients from a linearized gLV system, an approach popularized by Stein et al. 2013^42^, using regularized regression techniques. Specifically, we compared unregularized linear regression, L2-penalized ridge regression, L1-penalized lasso regression, and elastic net regression. Additionally, we implemented a recently published Bayesian inference approach.^1^ This approach uses an advanced prior structure and hierarchical setup for regularization, which we implemented here as “MK-SpikeSeq” to be evaluated on MTIST. Following the schema presented in Fig. 4A, we tested all five inference tools, calculating an ES score for each of the 648 datasets representing a hypothetical microbiome study. In Fig. 4A, we show a representative workflow to rank two inference tools on a single data set: we compare the ground truth community matrix for MTIST dataset ID 200 to their inference results and calculate ES scores. The unregularized linear regression outperformed the L2-penalized ridge regression in this example because there were 30 more net correctly inferred interactions using linear regression, resulting in a 0.18-point difference in ES score (0.70 vs 0.52).

Comparing the five inference algorithms tested on the entirety of MTIST (Fig. 4B) showed that linear regression and the regularized approaches generally performed similarly. For the 3- and 10-species communities (Fig. 4B), all inference algorithms outperformed the null model, showing that they were able to learn correct microbial interactions from the MTIST datasets. However, while all algorithms performed significantly better than the null-model on the 100-species community data (Fig. 4C, top), inference results were poor across all tested algorithms, with median ES scores of 0.52, 0.52, 0.52, and 0.51 for linear regression, ridge regression, elastic net regression, and MK-SpikeSeq, respectively, indicating only few interactions were identified correctly. To simulate stable ecosystems, the majority (9,843 out of 10,000, or > 98%) of the interaction coefficients in the 100-species community are small in magnitude^27^ (between − 0.25 and 0.25) compared to only ~ 69.97% (627 out of 900) and ~ 4.17% (3 out of 72) in the 10- and 3-species case, respectively. We hypothesized that ES scores were low because many of these weak interactions were overfit on the noisy simulation data, and thus inferred incorrectly. Thus, we calculated the ES score only on interactions with large magnitude in the corresponding community matrix ground truth (defined by an interaction coefficient where |*β*_*ij*_| > 0.25). Indeed, evaluating inference tool performance in this way (Fig. 4C, bottom) yielded for linear regression, ridge regression, elastic net regression, and MK-SpikeSeq median adjusted ES scores of 0.73, 0.77, 0.73, and 0.74, respectively, with correctly inferred *pairs* of interaction coefficients of 67% in case of ridge regression, 65% in case of MK-SpikeSeq, the top two performing algorithms. Overall, ridge regression significantly outperformed the other inference methods used in this computational experiment. We considered that, in practice, the unknown ground truth prevents such result filtering by interaction strength when analyzing real-world data. Reassuringly, however, we find that simply filtering inference results by magnitude directly, focusing only on large inferred coefficients also improves ES scores (**Figure S2**).

**Figure 5:**
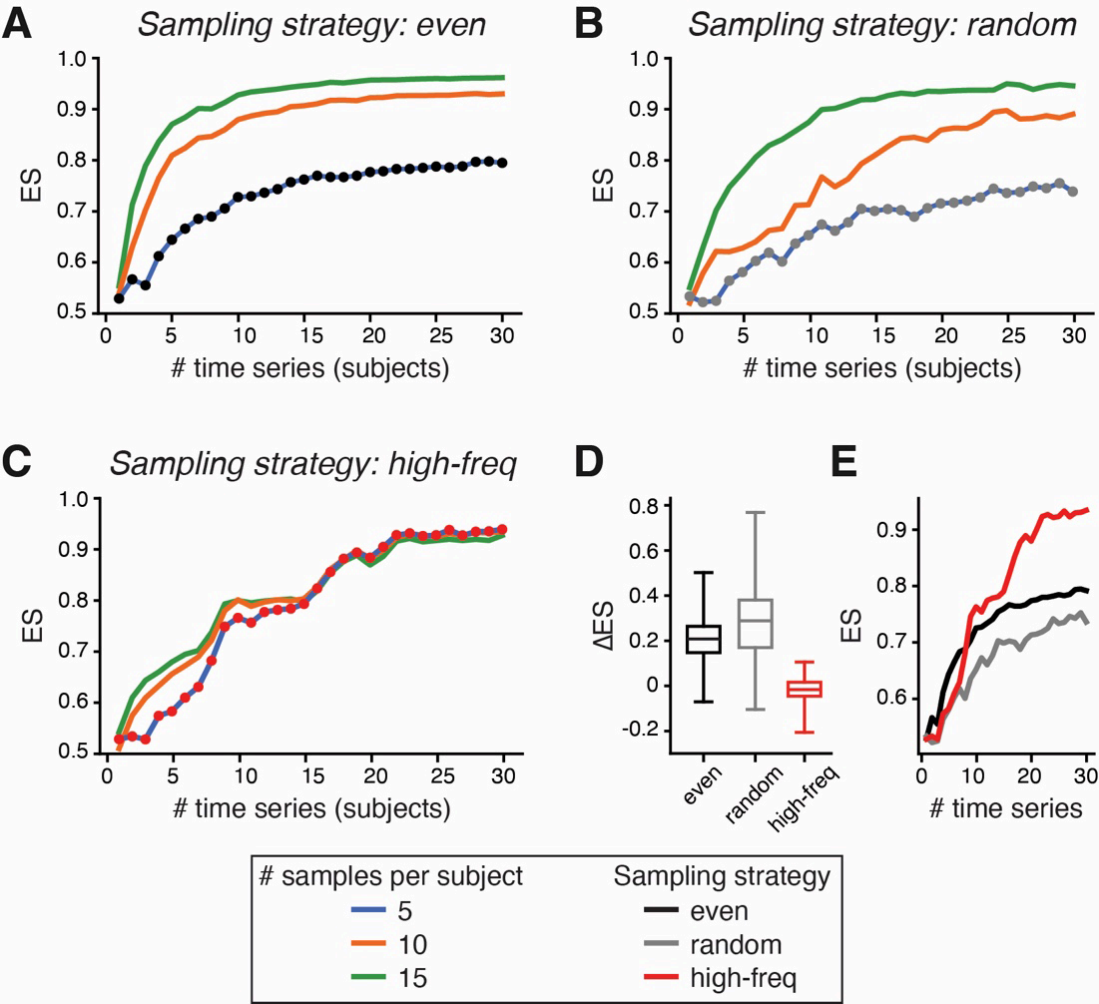
MTIST enables systematic comparison of study designs. ES score comparison using unregularized linear regression of data from 10-species ecosystems when 5, 10, or 15 samples per subject are recorded evenly along a fixed time period (A “even”), randomly during the simulated study period (B “random”), or with a strategy where samples are collected with the highest implemented frequency possible starting at a random point along the simulated study period (C, “high-freq”), and when the number of time series (i.e. simulated study subjects) are varied. D ES scores generally increase with more samples per subject; changes in ES scores calculated using 5 vs. 15 samples from 30 timeseries (subjects). E If only 5 samples per subject are available, high-frequency sampling leads to higher ES scores.

Beyond ranking inference tools, MTIST comprises simulations of different study designs, enabling a comparison of different sample collection strategies for ecosystem inference in the idealized setting of simulated microbiomes. During a fixed simulated time period and with a fixed number of species abundance values recorded for inference (“samples”), such “samples” are recorded in MTIST1.0 in one of three ways along the time course: ad hoc, i.e. randomly with varying time differences between samples (“random”), evenly distributed (“even”), or sequentially with maximum temporal frequency (“high-freq”) of allowed within the MTIST data, simulating e.g. “daily” stool collection (Fig. 1D). With this at hand, we can systematically assess inference performance in different study designs when either the number of samples per simulated time series (i.e. the number of samples collected from one individual subject), or the number of time series analyzed (i.e. the number of subjects studied) are altered. As expected, both the number of samples per time course and the number of time courses analyzed increase the ES score (Fig. 5A–C, linear regression performed on 10-species ecosystems in MTIST1.0). As the number of time courses analyzed is increased, ES scores appear to converge, albeit to different maximum levels depending on the study design. Interestingly, while the number of samples collected per time course clearly improved inference when samples were collected at random or evenly (Fig. 5A and B, respectively), in the setting where samples were collected sequentially with high temporal frequency, increasing the number of samples collected per time course resulted in less striking improvements (Fig. 5C). The high-frequency study design performed particularly well relative to the other designs when only 5 samples were available for each time course; in this setting, our model predicts that collecting samples with a high frequency improves the chance of capturing meaningful dynamics that inform successful inference.

## Discussion

We have presented a large set of *in silico* time series data (MTIST1.0) and a corresponding evaluation score, the ecological sign (ES) score, to be used by microbial ecosystem inference algorithms evaluated on the 648 datasets compiled for MTIST. We demonstrated the utility and application of this resource by evaluating different implementations of gLV inference algorithms. Using the ES score, we demonstrated that each implementation can achieve significant learning of ecosystem rules in simple ecosystems. Understanding these interactions could reveal strains that may combat the invasion of human gut pathogens,^43^ improve the predictability of FMTs,^26^ or accelerate the development of “evolutionary medicine” approaches to limit pathogen growth by designing and/or introducing competitor species.^57^

While other models are used in theoretical ecology, gLV models have dominated the study of microbiome dynamics, and form the basis of most inference algorithms. Therefore, MTIST represents an idealized scenario for these algorithms since the model of microbial ecology that they assume matches the data-generating process closely, unlike real-world data with unknown, more complex rules. Despite our favorable set up of a simplistic ecology, we find that none of the tested algorithms achieved high ES scores in the large simulated ecosystems that comprised 100 species. Ecological theory predicts that all else being equal, stronger interactions between species can lead to feedbacks that prevent ecosystem stability. Therefore, most interactions between species need to be set to zero or must be weak when simulating large ecosystems. We find that when focusing on the sparse strong interactions, the inference was indeed successful, leading to all tested algorithms identifying key interaction pairs correctly. Our results demonstrate that despite regularization, which can induce sparsity and lead to more conservative inference results, the data was overfit. However, we showed that simply filtering inference results by magnitude improved the calculated ES scores, meaning that the larger coefficient pairs tend to be more trustworthy. This validates the choice often made in experimental or observational work where strong inferred interactions are the focus for discussion or follow-up work. Indeed, reassuringly, in a recent study using gLV inference, the strongest inferred interaction pairs identified from microbiome time series data yielded consistent observations in validation experiments.^1^

The MTIST platform represents a versatile tool to test difficult to intuit choices in microbiome research in a simplified setting. For example, we investigated the ideal strategy for sample collection in a hypothetical microbiome study and revealed that when only few samples can be collected per individual subject, these are best collected with high temporal frequency. Intuitively, this maximizes the chances of capturing dynamics that gLV-inference approaches require. As a corollary, this also suggests an optimal study design when a perturbation to the microbiome is known to occur, e.g. a scheduled dietary change or antibiotic intervention: in such a case, our results suggest that sampling with high frequency directly after the external perturbation would yield the maximum information on between-species interactions. Additionally, MTIST is extendable to new research questions. The entire MTIST codebase is publicly available on GitHub, including the procedures to produce bespoke MTIST datasets with customized simulation parameters. Using this code, experimenters can create *in silico* data matching their experiments to probe theoretical performance of their inference tools applied to their specific study design.

Our results nevertheless highlight the magnitude of the remaining challenge posed by learning ecological rules from metagenomic data, and that there is ample room for improvement with novel inference tools. Taken together, we are providing a unified platform to develop better approaches to understand microbiome ecology.

## Methods

### Simulation framework

MTIST1.0 is available as a compressed archive in the supplement and online via github at github.com/jsevo/mtist. We first developed a generalized Lotka-Volterra-based simulation framework for microbial time series, then generated datasets for 3-, 10-, and 100-species microbial communities using a wide range of simulation conditions that aim to mimic human microbiome time series data. We used the Python programming language to simulate microbial abundances for 25 days in 100 discrete “6-hour” steps, introducing non-differentiable biological perturbation at the end of each of these steps (**Figure S1**). Our implementation of perturbations allows for the introduction of additional sources of abundance changes (e.g., migration events) at custom-chosen time points. This methodology is therefore highly customizable. For example, beyond a stochastic differential equation-based implementation of noisy gLV, it can allow the implementation of a specific migration event at a specific time. While this was not explicitly performed to generate MTIST v1.0, our code platform therefore enables modeling the administration of an FMT at a specific time point. In between perturbation events, the differential equations are then solved numerically using the scipy package for the Python programming language, yielding “master time series”, i.e. the complete time trajectory of the ecosystem invisible to an experimenter, for each initial species abundances (Fig. 1D). We then sampled hypothetical microbiome studies from these master time series, resulting in the 648 datasets that form MTIST1.0. We provide full explanation of simulations, ground truth construction, and scoring system details in the **Supplementary Methods**.

### Inference tool implementation

We used linearized difference equations derived from gLV systems as a parameterized mathematical model representing microbial ecosystems driven by pairwise interactions.


$$\frac{\Delta ln\left(X_{i}\right)}{\Delta t}=r_{i}+\sum_{i=0}^{N}\;\beta_{ij}X_{j}$$

In these equations, change in the log difference of a focal species (j) between two time points, $\Delta ln\left(X_{i}\right)$, is weighted by the length of time between two time points (Δt) and predicted using the sum of geometric means of all other species $\left(X_{j}\right.$ for $\left.j\in N\right)$ in the community. These other species’ abundances are multiplied by an interaction coefficient, $\beta_{ij}$ When i=j, $\beta_{ij}$ represents the self-interaction coefficient. Lastly, $r_{i}$ is the growth rate for species *i*.

We used the LinearRegression, LassoCV, RidgeCV, and ElasticNetCV methods from the linear_model module in the scikit-learn package for the Python programming language to estimate $\Delta ln\left(X_{i}\right)$ using non-penalized, L1-, L2-, and L1-/L2-penalized regression, respectively. Lastly, we also implemented MKSeqSpike (Rao et al. 2021) by adapting the author’s publicly-available code.

### Study design analysis

Using the framework provided by MTIST, we evaluated the effect of experimental design on the quality of inference results. More specifically, we focussed on the effects of varying the number of samples collected from a given patient (number of points in a given time series) and the number of patients involved in a study (number of time series in a dataset). We consider the effect of experimental design over a wide range of possible microbiome communities by extending MTIST with extra datasets generated from 50 different ground truths of 10-species communities, over which results were averaged. These ground truths were generated from uniform distributions for growth rates and normal distributions for interspecies interactions, with negative intraspecies interactions, selecting only for ground truths that allow for stable communities which do not inherently lead to species extinction in the absence of non-differentiable noise. For these 50 new ground truths, we simulate experiments with 5, 10, and 15 points per time series, also varying the total number of time series in a dataset from 1 to 30, in order to observe the effect in the ES scores obtained from unregularized linear regression (Fig. 5).

We analyze how the obtained ES score changes while filtering interactions by their strength, i.e. their absolute values (**Figure S2**). Stratifying the interactions by the percentile rank of their magnitudes with respect to the distribution of interaction strengths over a given community, we averaged results over the different simulated communities. For different percentiles of interaction strength, we calculated the ES score obtained considering only interactions stronger than a focal percentile.

### Using MTIST1.0

The MTIST framework has minimal requirements for an algorithm’s application programming interface; it only expects a community matrix (species-by-species interaction coefficients) that can be compared to a ground truth. We propose that researchers looking to use MTIST to benchmark their inference algorithm’s performance run at least two tests: (1) can the inference algorithm beat unregularized linear regression in the 3- and 10-species case; (2) can the inference algorithm accurately capture strong interactions in the 100-species case, as our tested algorithms struggle on this task.

## Data Availability

The MTIST dataset from this study can be found in the mtist repository on GitHub (github.com/jsevo/mtist). The code to generate our data is included in the same repository, together with examples in the form of Jupyter notebooks.
